# Research Progress on Mesenchymal Stem Cells for the Treatment of Diabetes and Its Complications

**DOI:** 10.1155/2023/9324270

**Published:** 2023-04-24

**Authors:** Jiarui Zhang, Yongqin Zheng, Lichenlu Huang, Jundong He

**Affiliations:** ^1^Medical School, Kunming University of Science and Technology, No. 727 Jingming South Road, Kunming 650000, Yunnan, China; ^2^Department of Endocrinology and Metabolism, The First People's Hospital of Yunnan Province, The Affiliated Hospital of Kunming University of Science and Technology, No. 157 Jingbi Road, Kunming 650000, Yunnan, China

## Abstract

Diabetes mellitus (DM) is a chronic disease that threatens human health. Although many drugs are available to treat DM, various complications caused by DM are unavoidable. As an emerging treatment for DM, mesenchymal stem cells (MSCs) have shown many advantages and are gradually gaining public attention. This review summarizes the clinical studies on the use of MSCs to treat DM and the potential mechanisms of complications such as pancreatic dysfunction, cardiovascular lesions, renal lesions, neurological lesions, and trauma repair. This review focuses on the research progress on MSC-mediated secretion of cytokines, improvements in the microenvironment, repair of tissue morphology, and related signaling pathways. At present, the sample sizes in clinical studies of MSCs in treating DM are small, and there is a lack of standardized quality control systems in the preparation, transportation, and infusion methods, so we need to conduct more in-depth studies. In conclusion, MSCs have shown superior potential for use in the treatment of DM and its complications and will hopefully become a novel therapeutic approach in the future.

## 1. Introduction

Diabetes mellitus (DM) is a group of metabolic diseases characterized by chronic hyperglycemia and are caused by multiple etiologies and genetic and environmental factors. The pathological mechanism involves insufficient secretion and/or defective effects of insulin. Based on its pathogenesis, DM is mainly divided into two types: insulin-deficient type I (T1DM) and insulin-resistant type II (T2DM). It is expected that there will be 693 million DM patients worldwide by 2045 [1, 2]. The pathogenesis of DM is still unclear and may involve a variety of factors, such as family history, obesity, poor diet, and lack of exercise. DM can lead to a variety of complications, including ketoacidosis, cardiovascular disease, renal failure, peripheral neuropathy and retinopathy, and even lower limb amputation [3]. These complications seriously affect the health of patients with DM and even lead to death. Therefore, the goal of clinically treating DM patients should not only include glycemic control, but the longer-term goal should be to prevent and delay the occurrence and development of complications based on maintaining homeostasis.

Mesenchymal stem cells (MSCs), a heterogeneous subpopulation of stromal stem cells, can be isolated from a variety of tissues, such as bone marrow, peripheral blood, umbilical collagen, dental pulp, and adipose tissue. Depending on their tissue origin, MSCs can be broadly classified as umbilical cord-derived MSCs (UC-MSCs), bone marrow-derived MSCs (BM-MSCs), adipose-derived MSCs (AD-MSCs), Wharton's Jelly-derived MSCs (WJ-MSCs), placenta-derived MSCs (PL-MSCs), and breast milk-derived MSCs (Br-MSCs). The diversity of tissue sources makes MSCs ethically superior to embryonic stem cells (ESCs) [4]. MSCs have lower immunogenicity, have self-renewal, immunomodulatory, and anti-inflammatory properties, and have been widely used in the treatment of various immune diseases, including DM and its complications [5]. The potential mechanisms by which MSCs act include the regeneration and survival of islets, homing to target organs, differentiation, and systemic immune regulation [6]. However, some factors also limit the therapeutic efficacy of MSCs, such as differences in cell characteristics, regimens used, doses used, and infusion methods. Therefore, we urgently need a standardized quality control system and more clinical application data to make this treatment method available to patients as soon as possible [7].

## 2. Clinical Research Progress on MSCs as a Treatment for DM

### 2.1. Clinical Studies on MSCs for the Treatment of T1DM

We summarized 3 recent clinical study developments on the clinical application of MSCs for the treatment of T1DM. In a phase I clinical trial, Madani et al. treated 4 patients with T1DM by using PL-MSCs. The 3-month follow-up revealed a decrease in hemoglobin A1c (HbAc1) and an increase in C-peptide (CP) levels in 2 patients. Antiglutamic acid decarboxylase (GAD) antibody levels were decreased in 3 patients, and antizinc transporter protein 8 (ZnT8) antibody levels were decreased in all patients[8]. In addition, in a 3-month trial, 8 patients with T1DM were treated with AD-MSCs, and 5 patients in the control group were treated with insulin only. At 3 months, the CP level in the trial group was 211.20 ± 100.42 ng/ml (control group 106.05 ± 47.25 ng/ml), the insulin requirement was 0.22 ± 0.17 IU/kg (control group 0.61 ± 0.26 IU/kg), and HbAc1 was 6.47 ± 0.86% (control group 7.48 ± 0.52%) [9]. In a randomized, double-blind, phase I/II clinical trial, 11 patients with T1DM were treated with BM-MSCs, and the percentage of HbAc1 was significantly reduced at 12 months. Cytokine interleukin 4 (IL-4) and interleukin 10 (IL-10), which have anti-inflammatory effects, were significantly increased, while tumor necrosis factor-alpha (TNF-*α*), which has proinflammatory effects, was significantly decreased [10]. Taken together, these results showed that the use of MSCs reduced HbAc1 and insulin requirements in T1DM patients, decreased the levels of signature antibodies against GAD and ZnT8 in T1DM patients, increased CP levels, and enhanced systemic anti-inflammatory capacity.

### 2.2. Clinical Studies on MSCs for the Treatment of T2DM

Clinical studies on the use of MSCs to treat T2DM have received much attention. In 2009, investigators used BM-MSCs to treat 10 patients with T2DM and found a 50% reduction in insulin use in 7 patients over 6 months, a decrease in HbAc1 from 8.1 ± 0.2% to 7.3 ± 0.4%, and a significant increase in fasting CP levels [11]. A study by Nguyen et al. using autologous BM-MSCs to treat patients with T2DM showed a significant reduction in HbAc1 levels and a decrease in fasting blood glucose levels from 8.5 ± 3.5 mmol/L at baseline to 6.8 ± 2.4 mmol/L over 12 months in some patients [12]. The same study also confirmed that, after 6 months of treatment with autologous BM-MSCs, some T2DM patients had a more than 50% reduction in insulin requirements from baseline and HbAc1 levels less than 7.0% [13]. In a single-center, double-blind, randomized phase II clinical trial, 45 patients with T2DM were intravenously administered UC-MSCs. Twenty percent of patients had HbAc1 less than 7.0% after 48 weeks, daily insulin requirements were reduced by more than 50%, and five patients were off insulin at 8–24 weeks, but there was no significant improvement in fasting CP levels [14]. In conclusion, these results show that MSCs can effectively improve several clinical indicators, such as HbAc1, fasting blood glucose, and insulin use, in patients with T2DM, showing good application prospects.

## 3. Studies on the Mechanism of MSCs for the Treatment of DM and Its Complications

The clinical studies in the previous section demonstrated the potential of MSCs in the clinical management of DM. This section focuses on their potential mechanisms.

### 3.1. MSC-Mediated Improvements in Pancreatic Function and Insulin Resistance in DM

#### 3.1.1. MSC-Mediated Improvements in Pancreatic Function in DM

**Figure 1:**
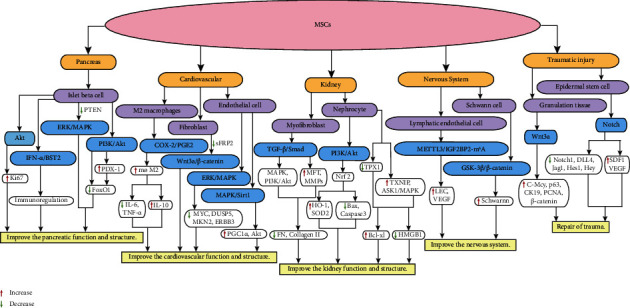
MSCs regulate different signaling pathways in individual target tissues or cells, which in turn regulate the secretion levels of cytokines and ultimately improve the function and structure of the target tissues.

The pancreas is a gland that plays an important role in metabolism. Pancreatic volume is significantly reduced in DM patients, and the structure and function of islet *β*-cells are severely disrupted, affecting systemic glucose metabolism [15]. MSCs can improve the pancreatic structure and function in DM patients by inducing the differentiation of MSCs into islet *β*-cells, alleviating insulin resistance (IR) and systemic immune modulation. Studies have shown that a high-glucose environment reduces pancreatic *β*-cell activity, but when BM-MSCs and islet *β*-cells were cocultured, there was a significant increase in islet *β*-cell activity, a significant increase in cell proliferation-associated protein Ki67, and an increase in insulin secretion. These effects were mediated by the protein kinase B (AKT) signaling pathway [16]. Bao et al. [17] induced high tissue inhibitors of matrix metalloproteinase (TIMP)-1 expression and injected the cells into T1DM mice. The mice had reduced blood glucose levels, improvements in the pancreatic structure and function, and showed clearer nucleoplasmic boundaries. Modifying MSCs to show unique advantages, this change may become a way to optimize the function of MSCs. One study used BM-MSCs with high expression of human telomerase reverse transcriptase to treat nude mice with the partially resected pancreases. The results showed a significant decrease in proinflammatory cytokines such as interferon-*γ* (IFN-*γ*) and TNF-*α* and a significant increase in insulin synthesis [18]. Further studies revealed that phosphorylation levels of the phosphatidylinositol 3-kinase/AKT (PI3K/AKT) signaling pathway were downregulated, while pancreatic duodenum homeobox-1 (PDX-1) expression was elevated, which affected the expression of FoxO1, a key regulator of pancreatic *β*-cells, and thus promoted islet *β*-cell regeneration (Figure 1) [18]. In addition, Br-MSCs can improve the islet microenvironment and enhance islet cell function by regulating endoplasmic reticulum stress, inflammation, and apoptotic signaling pathways [19]. The mechanisms include MSC-mediated upregulation of regenerative markers, anti-inflammatory markers, and antiapoptotic markers and downregulation of endoplasmic reticulum stress markers (ATF3, ATF6, CHOP, JNK, XBP1, and BIP) and an autophagy inhibitor (mTOR) [18]. T1DM mice with pancreatic damage were treated with wild-type P53-induced phosphatase 1 (Wip1)-knockout MSCs [20]. The results showed that this treatment was less effective and less immunosuppressive, whereas MSCs with normal expression of Wip1 upregulated bone marrow stromal cell antigen 2 (BST2) and IFN-*α* expression. These results may suggest that Wip1 affects the immunomodulatory function of MSCs in T1DM patients by targeting the IFN-*α*/BST2 pathway[20].

Several problems have also emerged with the current treatment with MSCs, including the high number of islet beta cells needed to restore normoglycemia, the low survival rate of cells after transplantation, and the rapid decline in islet function viability after transplantation. In contrast, in a coculture system using MSCs mixed directly with islet *β*-cells in suspension, the survival rate of islet *β* cells can reach 50% [21, 22]. Direct injection of MSCs into the pancreatic site increases the number of replicating islet cells and islet areas in vivo through physical contact. Physical contact also protects min6 cells derived from streptozotocin in vitro via AKT and extracellular regulated protein kinase (ERK) pathways [23]. In addition, increasing attention is being paid to MSC exosomes and cellular secretions. MSC exosomes contain a variety of bioactive substances, such as lipids, proteins, and RNAs, which can regulate the biological activity of target cells through membrane fusion or endocytosis[24]. MSC exosomes significantly increased the survival of pancreatic islet *β*-cells under hypoxic conditions and downregulated the expression of p38 AMP‐activated protein kinase (MAPK) and endoplasmic reticulum stress-associated proteins in *β*-cells [25]. When treated with MSC exosomes, mouse pancreatic tissue showed increased expression of genes associated with tissue regeneration pathways, including REG2, Reg3, and AMY2B [26].

#### 3.1.2. MSC-Mediated Improvements in Insulin Resistance in DM

In T2DM-related studies, some investigators showed that early intervention with BM-MSCs significantly reduced insulin levels and blood glucose levels and improved the abnormal expression of IR-related proteins in T2DM rats [27]. Treatment of T2DM rats with skeletal muscle injections of UC-MSCs resulted in significant improvements in IR in rats. This regulatory mechanism may involve the inhibition of inflammation by PTEN genes, which in turn regulate the balance between the PI3K/Akt and ERK/MAPK signaling pathways [28]. The human umbilical cord mesenchymal stem cell-derived small extracellular vesicle also improved serum HbA1c levels and upregulated alanine aminotransferase and alkaline phosphatase levels in T2DM rats and attenuated structural damage to the pancreas, kidney, and liver[29]. Phosphorylation of insulin receptor substrate 1 and AKT in T2DM rats was restored by intravenous injection of MSC exosomes, which promoted glucose transporter protein 4 expressions in the muscle, increased hepatic glycogen storage in the liver, maintain glucose homeostasis, and improved IR [30].

#### 3.1.3. Adverse Effects of MSC Adjuvant Therapy after Islet Transplantation

In addition, after islet transplantation, the instant blood-mediated inflammatory reaction, hypoxic and ischemic injury, and immune response will affect the survival of the patient. There is an increased risk of infection and serious disease and even a decline in graft function over time[31]. However, adjuvant therapy with MSCs after islet transplantation decreased the expression of inflammatory markers, increased the expression of immune tolerance markers, and decreased rejection, effectively enhancing islet survival [32]. Treatment with AD-MSCs effectively reduced inflammation and reconstituted islet vascularization in islet-transplanted DM mice, as well as upregulated the expression of angiogenic factor markers such as hepatocyte growth factor (HGF) and angiopoietin-1 (Ang-1) [33].

In conclusion, MSCs have shown great advantages in the treatment of T1DM or T2DM and as an adjuvant therapy after islet transplantation. MSCs can protect pancreatic tissue, maintain the islet *β*-cell structure and function, promote insulin secretion, and improve IR by affecting different signaling pathways, attenuating the inflammatory response and modulating the immune response.

### 3.2. MSC-Mediated Improvements in DM Combined with Cardiovascular Disease

Cardiovascular disease is a common complication in patients with DM. Cardiovascular complications have been reported in more than 50% of DM patients, and hyperglycemia is considered to be an important factor in structural alterations and dysfunction in cardiomyocytes and endothelial cells [34]. A study by Jin et al. [35] showed that MSCs were effective in improving diabetic cardiomyopathy (DCM). After treatments with AD-MSCs, LVEF and FS, which reflect the systolic function of the heart, were reduced in DCM rats, while E/A, which reflects the diastolic function of the heart, was significantly increased. Further studies revealed that MSCs increased the polarization of M2-type macrophages, inhibited the production of proinflammatory factors such as interleukin 6 (IL-6) and TNF-*α*, and increased the level of anti-inflammatory factor IL-10, reducing myocardial inflammation through the cyclooxygenase-2/prostaglandin E2 (COX-2/PGE2) signaling pathway (Figure 1). In addition, the COX-2/PGE2 pathway decreased the levels of transforming growth factor-*β* (TGF-*β*), collagen I, and collagen II, inhibited fibroblast proliferation and collagen synthesis, and attenuated cardiac fibrosis [35]. Treatment of DCM rats with resveratrol (RSV)-pretreated BM-MSCs attenuated cardiomyocyte injury and fibrosis in rats [36]. The potential mechanism involved the downregulation of the Wnt3a/*β*-catenin signaling pathway by inhibiting the expression of secreted frizzled-related protein (sFRP2), which in turn alleviated pathological remodeling of the heart and improved cardiac function [36]. Pretreatment with RSV also upregulated the expression of chemokine receptor CXCR4 in AD-MSCs, inhibited damage to MSCs in a high-glucose environment, and promoted the migration of MSCs to target organs [37]. RSV pretreatment of AD-MSCs activated the MAPK/sirtuin 1 (Sirt1) signaling pathway and increased peroxisome proliferator-activated receptor-*γ* coactivator (PGC)-1*α* and Akt expression, which in turn inhibited oxidative stress-induced endothelial cell apoptosis and reduced the risk of heart failure [37, 38].

WJ-MSCs were shown to significantly ameliorate endothelial cell dysfunction, inflammation, and oxidative stress in rats with DM combined with vascular lesions [39]. MSCs successfully homed to the small vessel and aortic endothelium, leading to significant reductions in systolic blood pressure (SBP) and aortic pulse wave velocity (PWV). This regulatory mechanism is associated with the upregulation of endothelial NO synthase (eNOS) expression and reduced levels of reactive oxygen species (ROS), vascular endothelial growth factor (VEGF), serum endothelin-1 (ET-1), and angiotensin II (Ang-II) [39]. Another study showed that UC-MSCs could inhibit aberrant phosphorylation of the ERK/MAPK signaling pathway and ameliorate the overexpression of MKNK2, ERBB3, MYC, and DUSP5-related genes, thereby protecting the vascular endothelium against DM injury[40]. Systemic immunomodulation and modification of genes is one way in which MSCs and their exosomes work, through which the levels of relevant inflammatory factors and hormones in the body are improved, and protects against cardiovascular damage from a high-glucose environment. Metformin is a common hypoglycemic drug, and one study showed that metformin, a MAPK activator, inhibited the improvements in DCM induced by BM-MSCs [41]. Metformin interfered with the homing and differentiation of BM-MSCs to the heart. However, the inhibition of MAPK increased CXCR4 levels and improved the survival of BM-MSCs under high-glucose conditions, allowing BM-MSCs to better attenuate myocardial fibrosis and increase vascular density [41].

In conclusion, the combination of cardiovascular complications with DM seriously affects the health and life of patients, and MSCs have good efficacy in improving cardiovascular status. On the one hand, MSCs reduce inflammation and inhibit fibrosis. On the other hand, MSCs also show some advantages in improving endothelial dysfunction and decreasing oxidative stress.

### 3.3. MSC-Mediated Improvements in DM Combined with Nephropathy

Diabetic nephropathy (DN) is a serious complication in DM patients. This condition is characterized by lipotoxicity-induced apoptosis and inflammatory cell infiltration, which is characterized by macrophage activation [42]. UC-MSCs were shown to significantly improve renal function, reduce local and systemic inflammatory cytokine levels, and attenuate the infiltration of inflammatory cells into renal tissue in rats with DN [43]. The mechanism involves shifting the polarization of macrophages from a proinflammatory M1 phenotype to an anti-inflammatory M2 phenotype by inhibiting tumor necrosis factor receptor-associated factor 6 (TRAF6) and the signal transducer and activator of transcription (STAT1) signaling pathways [43]. UC-MSCs improve oxidative damage and apoptosis in the kidneys of DN rats. UC-MSCs can activate the PI3K/Akt signaling pathway, causing nuclear factor erythroid 2-related factor 2 (Nrf2) to translocate to the nucleus and promote downstream factor expression, which in turn inhibits proapoptotic proteins such as Bax and caspase3 and upregulates heme oxygenase-1 (HO-1) and superoxide dismutase 2 (SOD2) levels [44]. However, the specific way in which UC-MSCs affect the PI3K/Akt signaling pathway was not demonstrated. In addition, it has been shown that UC-MSCs significantly reduce the levels of the proinflammatory factors such as IL-6, interleukin 1 beta (IL-1*β*), and TNF-*α* and the profibrotic factor such as TGF-*β* in the kidney and blood in DN rats and improve renal interstitial fibrosis [45]. Likewise, we believe that it is necessary to further investigate the relationship between pharmacokinetics and the efficacy and toxicity of UC-MSCs in diabetic nephropathy. This will reveal their potential mechanisms of action and provide a basis for clinical use. UC-MSCs have also been shown to reduce fibronectin (FN) and collagen I deposition by inhibiting the TGF-*β*/Smad signaling pathway, triggering myofibroblast transdifferentiation (MFT) and PI3K/Akt, MAPK signaling pathway-mediated cell proliferation, and increasing the levels of matrix metalloproteinases (MMPs) (Figure 1) [46]. Angiotensin-converting enzyme 2 (ACE2)-modified MSCs can also inhibit the TGF-*β*/Smad signaling pathway and reduce the levels of Ang-II, thereby inhibiting the renin-angiotensin system (RAS) and reducing glomerular fibrosis [46, 47].

Hyperglycemia leads to oxidative stress-mediated apoptosis and fibrosis in human renal cortical proximal tubule epithelial cells (HK-2 cells) by inducing mitochondrial ROS, which activates the MAPK signaling pathway. The overexpression of HSP70-interacting protein (CHIP) in MSCs rescued mitochondrial oxidative stress and apoptosis under high-glucose stress [48]. Lin et al. [49] found that BM-MSCs significantly reduced the expression of toll-like receptor 4/nuclear factor kappa B (TLR4/NF-*κ*B) and monocyte chemoattractant protein-1 (MCP-1) and attenuated glomerular and tubulointerstitial fibrosis deposition. BM-MSCs also activate the PI3K/AKT signaling pathway, promote tubular expansion, reduce glomerulosclerosis and proteinuria levels, and improve thylakoid expansion and podocyte loss [49, 50]. Another study showed that UC-MSCs promoted the expression of antiapoptotic protein Bcl-xl and inhibited the expression of high mobility group protein B1 (HMGB1) in the rat kidneys [43]. The potential mechanism involves UC-MSC-mediated attenuation of renal cell injury and proteinuria in DN rats by upregulating thioredoxin-interacting protein (TXNIP), downregulating thioredoxin 1 (TRX1), and activating the apoptosis signal-regulated kinase 1 (ASK1)/MAPK signaling pathway in the kidneys of DN rats [43]. In addition, it was shown that the injection of UC-MSCs into the renal arteries of DN rats significantly decreased apoptosis-related proteins (caspase-3, PARP, and Bax), fibrosis-related proteins (TGF-*β* and Smad3), autophagy-related factors (LC3B-I/II, Atg5, and beclin-1), and inflammation-related factors (MMP-9, TNF-*α*, and NF-*κ*B). Indicators of endothelial functional integrity (CD31 and vWF) and VEGF were significantly increased. MSC exosome-carried miR-125a directly binds to histone deacetylase 1, regulates ET-1 activation, inhibits glomerular thylakoid proliferation and renal fibrosis, and suppresses the expression of IL-6 and type I collagen [51]. It was found that MSCs with high expression of miRNA-let7c could selectively localize to damaged kidneys, upregulate miRNA-let7c gene expression, and attenuate renal fibrosis through exosome transfer [52].

In conclusion, the kidney is often injured in DM patients, and DN may progress to renal failure or even uremia. Renal structure and function gradually improved and normalized after treatment with MSCs. The mechanisms involved MSC-mediated reductions in inflammatory cell infiltration, inhibition of proapoptotic protein expression, and improvements in fibrosis and endothelial functional integrity.

### 3.4. MSC-Mediated Improvements in DM Combined with Neurological Lesions

DM combined with neurological complications can involve any part of the nervous system, including central nervous system complications, peripheral neuropathy, and autonomic neuropathy. The most common of these is diabetic peripheral neuropathy (DPN). This condition may be associated with an increase in inflammatory cells, abnormal cytokine expression, oxidative stress, bone marrow ischemia, and proinflammatory changes [53]. One study showed that endogenous BM-MSCs in T1DM rats showed dysfunction before neuropathy, which was characterized by abnormalities in morphology, proliferation, and survival [54]. This impairment may affect the protective effect of BM-MSCs on the nervous system. Treatment of DM rats with BM-MSCs restored the histomorphology and ultrastructure of normal neurons, with significantly enhanced neurofilament protein (NFP) expression [54, 55]. When BM-MSCs were induced with fluoxetine, their in vitro survival and paracrine properties were enhanced; the mRNA expression of brain-derived neurotrophic factor (BDNF), VEGF, and IL-10 were significantly upregulated, while IL-1*β* was significantly decreased [55]. The mechanism by which BM-MSCs improve DPN is by activating the glycogen synthase kinase-3*β* (GSK-3*β*)/*β*-catenin signaling pathway, enhancing the survival of Schwann cells and reducing neuronal damage [56]. MSC exosome treatment of DPN significantly reduced the response threshold to thermal and mechanical stimulation in diabetic mice and increased the number of nerve fibers, myelin thickness, and axon diameter within the sciatic nerve epithelium. M1-type macrophages were reduced, and M2-type macrophages were increased [57].

In addition, diabetic foot ulcers (DFUs) caused by distal nerve abnormalities and peripheral vasculopathy in the lower extremities are the most common cause of nontraumatic amputation in DM patients. It has been reported that 85% of patients with DM with DFUs have their limbs amputated, which severely affects their quality of life [58]. Treatment with AD-MSCs accelerates wound healing in mice with DFUs, improves the reepithelialization of injured skin, and increases vascular redistribution in traumatized tissue [59]. Mechanistically, AD-MSCs restore intraepidermal nerve fiber (IENF) density, reduce apoptosis in neurons and Schwann cells, promote angiogenesis, and reduce chronic inflammation in peripheral nerves [59]. DFU model rats were treated intravenously with UC-MSCs and showed a significantly higher wound healing rate (45.1% ± 1.8% vs. 26.7% ± 2.1%) on day 8 and a wound healing rate approaching 90% on day 16 [60]. Further studies revealed that UC-MSCs promoted epithelialization and collagen deposition, granulation tissue formation, vascular redistribution, and antiapoptotic cell death. The diffuse distribution of positive cells containing Ki-67, which is a cell proliferation-associated protein in traumatized tissue, is also an important factor in wound healing [60]. In DFU model mice, intravenous administration of AD-MSCs accelerated proliferation, migration, and lymphatic endothelial cell (LEC) generation mediated by the methyltransferase-like 3 (METTL3) signaling pathway [61]. The regulation of VEGF expression occurs through the METTL3/insulin-like growth factor 2 binding protein 2-N6 methyladenosine (IGF2BP2-m6A) pathway (Figure 1) [61]. Huang et al. [62] found that TNF-*α*, IL-6, and VEGF receptor 1 (FLT-1) induced exosome secretion from BM-MSCs, and further studies confirmed that exosomal RNA-21-5p promotes angiogenesis and facilitates ischemic tissue repair by upregulating VEGFR, AKT, and MAPK.

In conclusion, neuralgia, sensory loss, and foot ulcers caused by DM combined with neuropathy seriously affect patient quality of life. It is difficult to develop effective control of this class of complications through the regulation of blood glucose alone. However, treatment with MSCs can significantly improve neurological dysfunction and protect the histomorphology and ultrastructure of neurons while upregulating BDNF, VEGF, and anti-inflammatory factors to promote the healing of DFUs and the redistribution of trauma vessels.

### 3.5. MSC-Mediated Repair of DM Trauma

Wound repair is a dynamic process that is divided into four stages: hemostasis, the inflammatory response, epithelial and neovascularization, and the remodeling of connective tissue. It is well known that poor glycemic control in DM patients can lead to neuropathy and impaired blood circulation, and wounds often do not heal properly. When AD-MSCs were directly transplanted into the wounds of DM mice, the expression of collagen I, collagen III, alpha-smooth muscle actin (*α*-SMA), and platelet endothelial cell adhesion molecule-1 (CD31) in the wound tissue was significantly increased and the expression of IL-6 was significantly decreased, which in turn promoted wound healing and hair follicle regeneration [63]. The mechanisms underlying these effects are the direct differentiation of MSCs into fibroblasts and vascular endothelial cells in vivo, as well as the promotion of fibroblast growth and migration [63]. The expression levels of the angiogenesis-related factors such as VEGF, HGF, *α*-SMA, FLT-1, and VEGF receptor 2 (FLK-1) were significantly increased when BM-MSCs overexpressing interleukin 7 (IL-7) were transplanted subcutaneously into the wounds of DM rats, which in turn promoted wound healing [64]. Although the above studies showed good efficacy, their potential gene targets and signaling pathways of action need to be further explored. Overexpression of hematopoietic prostaglandin D synthase (HPGDS) in AD-MSCs was shown to reduce neutrophil and CD8 T-cell recruitment, promote M2 macrophage polarization, increase growth factor levels, and ultimately promote wound healing [63, 65]. However, a molecular mechanism for the reduction of HPGDS in diabetic trauma was not demonstrated. In addition, UC-MSCs increase the number of M2 macrophages, reduce the number of Ly6C-positive cells, and modulate the inflammatory response [65, 66]. Coculturing macrophages stimulated with human umbilical vein endothelial cells (HUVECs) in the UC-MSC-conditioned medium significantly increased the number of microtubules, migration rates, and chemotaxis in HUVECs [66]. Mechanistically, UC-MSCs upregulate IL-10 and VEGF expression in macrophages through the secretion of PGE2 and downregulate TNF-*α* and IL-6 levels [66]. Salidroside can regulate cellular metabolism and enhance cellular immunity. Pretreatment of MSCs cultured in high glucose with salidroside inhibited ROS levels in MSCs and improved their survival and migration [67, 68]. When salidroside-pretreated MSCs were transplanted into mouse wounds, significant upregulation of the key wound healing factors such as HO-1, HGF, and fibroblast growth factor 2 (FGF2) was observed [68]. WJ-MSCs were encapsulated in a PF127 hydrogel and sodium ascorbyl phosphate (SAP) complex and then transplanted onto T2DM rat wounds, where they reduced MSC apoptosis, decreased the number of M1 macrophages, and increased the number of M2 tissue healing macrophages, ultimately promoting dermal regeneration, collagen deposition, and neovascularization [69]. The use of drugs to stimulate MSC exosomes can lead to better therapeutic results than exosomes alone [70–72]. Exosomes from MSCs treated with atorvastatin promote endothelial cell proliferation, migration, tubular formation, and VEGF levels by activating the AKT/eNOS signaling pathway and upregulating miR-221-3p [71]. BM-MSCs treated with the same pioglitazone treatment had exosomes that promoted the angiogenic function of HUVECs through the PI3K/AKT/eNOS pathway, thereby accelerating the healing of diabetic wounds [72].

The relevant signaling pathways associated with the repair of DM trauma by MSCs include two major classes: Wnt and Notch (Figure 1) [73–75]. In the treatment of DM rat wounds with UC-MSCs, Wnt signaling pathway agonist Wnt3a effectively increased fibronectin deposition and capillary abundance in granulation tissue, but antagonist SFRP3 significantly slowed skin wound healing in DM rats [74]. The Wnt signaling pathway can upregulate *β*-catenin, the C-Myc gene, the p63 gene, cytokeratin 19 (CK19), and proliferating cell nuclear antigen (PCNA) expression, promoting the proliferation and differentiation of MSCs and reducing apoptosis [74]. DM rats with trauma were treated with AD-MSCs and platelet-rich plasma (PRP), which enhanced the proliferation and recruitment of epidermal stem cells (EPSCs), promoted reepithelialization and granulation tissue formation, and significantly increased the collagen area percentage, epidermal thickness, and vascular abundance [75]. Further studies showed that combination therapy downregulated the pathologically high expression of the Notch receptor such as Notch1, Notch ligands (DLL4 and Jag1), and Notch target genes (Hes1 and Hey1) and upregulated VEGF and stromal cell-derived factor-1 (SDF-1) expression [75].

In conclusion, slow wound healing, susceptibility to infection, and poor healing outcomes in DM patients have become major complications of DM, and the treatment of these complications is currently limited to the use of topical drugs to assist wound healing on an anti-infective basis. Wound grafting with MSCs can significantly accelerate healing, reconstruct the microvasculature, and effectively reduce infection. Therefore, MSCs have good prospects for treating DM wound repair.

## 4. Conclusions

MSCs have been of interest to many researchers because of their high proliferative capacity, potential for multidirectional differentiation, systemic immunomodulatory capacity, and low immunogenicity and immune rejection. Numerous studies have shown that MSCs have good efficacy in the treatment of DM and its complications. In this paper, we present the roles of MSCs from different sources in the treatment of pancreatic dysfunction, renal lesions, cardiovascular lesions, neurological lesions, and trauma repair and delineate various mechanisms, including the repair of tissue morphology, secretion of cytokines, and improvements in the microenvironment and signaling pathways. MSCs affect pathways involving PI3K/AKT, ERK/MAPK, COX-2/PGE2, GSK-3*β*/*β*-catenin, TGF-*β*/Smad, Wnt3a, and Notch (Figure 1). Different pretreatments can significantly improve the quality and quantity of MSCs in a high-glucose environment and effectively enhance their therapeutic effects. Moreover, this paper also summarized clinical studies of MSCs to treat DM. However, there are many shortcomings in the current stage of this field, such as small sample sizes and the lack of uniform quality control standards for MSCs. Therefore, we need to conduct large sample size and multicenter studies, as well as develop a standardized process for the preparation, transport, and infusion of MSCs. In conclusion, MSCs have shown great potential in the treatment of DM and its complications, and it is believed that MSC therapies can provide a new treatment option for patients in the near future.

## Data Availability

The datasets analyzed during the current study are available from the corresponding author upon reasonable request.
